# Use of electroencephalogram, gait, and their combined signals for classifying cognitive impairment and normal cognition

**DOI:** 10.3389/fnagi.2022.927295

**Published:** 2022-09-07

**Authors:** Jin-Young Min, Sang-Won Ha, Kiwon Lee, Kyoung-Bok Min

**Affiliations:** ^1^Veterans Medical Research Institute, Veterans Health Service Medical Center, Seoul, South Korea; ^2^Department of Neurology, Veterans Health Service Medical Center, Seoul, South Korea; ^3^Ybrain Research Institute, Seongnam-si, South Korea; ^4^Department of Preventive Medicine, College of Medicine, Seoul National University, Seoul, South Korea; ^5^Medical Research Center, Institute of Health Policy and Management, Seoul National University, Seoul, South Korea

**Keywords:** diagnosis, cognitive impairment, multimodal signals, EEG, kinematic gait analysis

## Abstract

**Background:**

Early identification of people at risk for cognitive decline is an important step in delaying the occurrence of cognitive impairment. This study investigated whether multimodal signals assessed using electroencephalogram (EEG) and gait kinematic parameters could be used to identify individuals at risk of cognitive impairment.

**Methods:**

The survey was conducted at the Veterans Medical Research Institute in the Veterans Health Service Medical Center. A total of 220 individuals volunteered for this study and provided informed consent at enrollment. A cap-type wireless EEG device was used for EEG recording, with a linked-ear references based on a standard international 10/20 system. Three-dimensional motion capture equipment was used to collect kinematic gait parameters. Mild cognitive impairment (MCI) was evaluated by Seoul Neuropsychological Screening Battery-Core (SNSB-C).

**Results:**

The mean age of the study participants was 73.5 years, and 54.7% were male. We found that specific EEG and gait parameters were significantly associated with cognitive status. Individuals with decreases in high-frequency EEG activity in high beta (25–30 Hz) and gamma (30–40 Hz) bands increased the odds ratio of MCI. There was an association between the pelvic obliquity angle and cognitive status, assessed by MCI or SNSB-C scores. Results from the ROC analysis revealed that multimodal signals combining high beta or gamma and pelvic obliquity improved the ability to discriminate MCI individuals from normal controls.

**Conclusion:**

These findings support prior work on the association between cognitive status and EEG or gait, and offer new insights into the applicability of multimodal signals to distinguish cognitive impairment.

## Introduction

With the growing proportion of the elderly population worldwide, the prevalence of dementia is rapidly increasing and is projected to reach 75 million by 2030 and 132 million by 2050 (Dua et al., 2017). Dementia is a clinical syndrome characterized by the loss of cognitive function, behavioral deterioration, and an impaired ability to perform everyday activities (McKhann et al., 2011). As the most common form of dementia, Alzheimer’s disease (AD) is thought to begin at least 10–20 years before the appearance of AD symptoms; however, there is a lack of modification therapy to delay the onset or alter its progressive course (Holtzman et al., 2011).

Early detection of cognitive decline is an important step in delaying the occurrence of mild cognitive impairment (MCI) and AD and maximizing illness control and improving the quality of life (Solomon et al., 2014). MCI is an intermediate stage between healthy aging and AD and is characterized by an objective cognitive decline in one or more cognitive domains without any significant impairment in activities daily living (Petersen et al., 2014). If MCI and AD are suspected, the clinical diagnosis mainly relies on neuropsychological tests, laboratory assessments, and brain imaging. However, their use has several limitations related to the low accuracy of cognitive tests, the limited validation and invasiveness of laboratory tests, and the high cost and low availability of the equipment. Recent advances in measuring biomedical signals have offered promising solutions to these limitations (Chen et al., 2020). Physiological signals (i.e., electrophysiological, biophysical, and biochemical signals) have been employed to assist in the clinical diagnosis or detection of impaired cognitive function (Chen et al., 2020). The present study focused on whether multimodal physiological signals can be used to assess the odds of cognitive impairment. To narrow the scope of multimodal physiological signals, we chose the specific signals of an electroencephalogram (EEG) and motor activity, with reference to a review of existing literature (Jeong, 2004; Bahureksa et al., 2017).

Electroencephalogram is a recording of electrical activity taken from the scalp, and reveals synaptic activity that is correlated with the brain state (Dauwels and Kannan, 2012). EEG is one of the most commonly used methods for monitoring and diagnosing abnormal brain function during a normal aging process (Cassani et al., 2018). Studies have shown that MCI and AD are associated with increased delta and theta activities, decreased alpha and beta activities, and a lesser mean peak frequency, although EEG dynamics are somewhat disputable (Huang et al., 2000; Jelic et al., 2000; Jeong, 2004). Further studies have proposed novel EEG-based methodologies for the diagnosis of patients with MCI or AD and normal individuals (Ahmadlou et al., 2011; Sankari et al., 2012; Amezquita-Sanchez et al., 2019). A high accuracy ranging from 83 to 99.3% distinguishing patients with AD from normal participants was reported using novel methodologies, such as the Katz fractal dimension, epoch-based entropy, and the integrated multiple signal classification and empirical wavelet transform (Ahmadlou et al., 2011; Houmani et al., 2015; Amezquita-Sanchez et al., 2019). Thus, these findings suggest that a specific pattern in EEG signals may be helpful in detecting individuals with cognitive impairment.

In addition, body movements are associated with several brain regions. A conventional measure is gait, which is an automatic motor activity regulated by subcortical and spinal regions. Although MCI criteria require relatively preserved daily activity functioning, individuals with MCI can experience gait dysfunction (Morris et al., 2016; Bahureksa et al., 2017). Under single-task conditions, several gait parameters (i.e., velocity, stride length, and stride time) were significantly discriminated for patients with MCI or AD in comparison with cognitively healthy individuals (Morris et al., 2016; Bahureksa et al., 2017). Furthermore, dual-task assessment (i.e., the concurrent performance of motor-cognitive tasks) revealed significant increases in stride time, step length, and step width variabilities in MCI vs. healthy controls (Boripuntakul et al., 2014; Bahureksa et al., 2017). Although these gait differences do not reflect a drastic decline in every function, gait assessment may be relevant for predicting cognitive decline and worsening of dementia (Allali et al., 2016; Lin et al., 2016; Bahureksa et al., 2017).

In this study, we investigated whether multimodal signals assessed using EEG and gait kinematic parameters could be used to identify individuals at risk of cognitive impairment. The rationale for the combined use of EEG and gait parameters is based on different approaches but consistent information that can help detect cognitive impairment. The EEG is an important physiological signal that reflects the activity of the cerebral cortex by collecting changes in the scalp electrode measurement (Jeong, 2004). Gait is a measure of motor abilities that reflects progressive brain dysfunction in aging and neurodegenerative diseases (Bahureksa et al., 2017). Both EEG and gait abnormalities are associated with the risk of cognitive decline (Huang et al., 2000; Jelic et al., 2000; Jeong, 2004; Morris et al., 2016; Bahureksa et al., 2017) and are considered useful for early identification of the presence of cognitive impairment (Ahmadlou et al., 2011; Sankari et al., 2012; Allali et al., 2016; Lin et al., 2016; Bahureksa et al., 2017; Amezquita-Sanchez et al., 2019). Modeling both EEG and gait parameters together might strongly predict cognitive impairment. To achieve this aim, we: (1) measured the EEG and gait among study participants; (2) compared the differences in the two measured signal types according to cognitive status (normal cognition vs. MCI); and (3) evaluated the ability of these multimodal signals (combined EEG and gait kinematic parameters) to distinguish individuals with MCI from those with normal cognition.

## Methods

### Study population

Individuals aged ≥60 years who visited the Department of Neurology at Veterans Health Service Medical Center (Seoul, South Korea) between March and December 2021 were the target population for the current study. The inclusion criteria were as follows: (1) those who complained of cognitive decline, (2) those who could independently complete clinical tests and questionnaires, and (3) those who agreed to participate in this study. The exclusion criteria were as follows: (1) a diagnosis of dementia (ICD-10: F00-F09, G30), (2) diagnosed with brain infarction, cerebral hemorrhage, or Parkinson’s disease, and (3) suffering from another serious disease (e.g., cancer or mental illness). The inclusion and exclusion criteria were evaluated by experienced neurological clinicians. The study protocols were approved by the Institutional Ethical Review Board of the Veterans Health Service Medical Center (IRB No. BOHUN 2021-02-024).

The participants underwent a health survey consisting of EEG and gait measurements, cognitive examinations, and questionnaires. The survey was conducted at the Veterans Medical Research Institute in the Veterans Health Service Medical Center. A total of 235 individuals volunteered for this study and provided informed consent at enrollment. Participants were requested to take part in the follow-up study. Of these, 15 participants subsequently dropped out of the study due to incomplete answers on disease history (*n* = 1), refusal to undergo EEG (*n* = 10), and abnormal gait (*n* = 4). After these exclusions, 220 individuals (93.6%) remained eligible for the current study.

### Electroencephalogram recording and processing

A cap-type wireless EEG device (MINDD SCAN, Ybrain Inc., South Korea) was used for EEG recording. The EEGs were recorded from 19 electrode sites (Fp1, Fp2, F7, F3, Fz, F4, F8, T7, C3, Cz, C4, T8, P7, P3, Pz, P4, P8, O1, and O2) with a linked-ear references based on a standard international 10/20 system. Participants underwent EEG recording at resting-state with eyes closed to measure endogenous pattern of brain activity of the target population. The sampling frequency and resolutions were 500 Hz and 24 bits, respectively.

For EEG signal processing, a 1 Hz high-pass filter and a 60 Hz notch filter were applied to remove eye and power noises. Independent component analysis was also used to eliminate eye-blink and muscle artifacts. For the analysis, over 3 min of artifact-free EEGs were selected from total 5 min of recorded resting-state EEG data. The relative power of six frequency bands, namely delta (1–4 Hz), theta (4–8 Hz), alpha (8–12 Hz), beta (12–25 Hz), high beta (25–30 Hz), and gamma (30–40 Hz) bands, was defined for further analysis of the target population.

### Gait analysis using three-dimensional motion capture equipment

Three-dimensional motion capture equipment was used to collect kinematic gait parameters using the NORAXON myoMOTION sensor (Scottsdale, AZ, United States), which is a wireless inertial measurement unit (IMC) system. The IMU sensor transmits human motion capture directly to the myoMOTION receiver to compute the angular changes of selected body segments. Using “Fusion algorithms,” the information from the accelerometer, gyroscope, and magnetometer was used to measure the three-dimensional rotation angles of each sensor in absolute space. The algorithm utilizes gyroscope and acceleration data from the foot-mounted IMU to identify the period when the foot is in the stance and swing phases via an on/off signal. The IMU was recorded at sampling rates of 100–200 Hz. The IMU data are mathematically combined, filtered, and processed using the myoMOTION software to quantify angular changes for joints, and the output is exported in Excel files (Alothmany et al., 2014).

For this study, the seven IMUl sensors were placed on the participants’ shoes (on the top of the upper foot), shins (frontal, on the tibia bone), thighs (frontal attachment to the lower quadrant of the quadriceps), and bony area of the sacrum, on both the left and right. Calibration was performed using the upright position to determine the value of the 0° angle in the studied joints. Participants were instructed to walk at their usual pace for 6 and 10 m. The kinematic parameters included the averaged signals for each stance phase and swing phase. The parameters of the stance and swing phases were the angles of hip abduction, hip flexion, hip external rotation, knee flexion, ankle abduction, ankle dorsiflexion, ankle inversion, pelvic tilt, pelvic obliquity, and pelvic rotation.

### Neuropsychological evaluation

Participants completed the brief version of the Seoul Neuropsychological Screening Battery (SNSB), named the SNSB-Core (SNSB-C; Kang et al., 2015). The SNSB-C is a comprehensive test that evaluates the level of cognitive function or impairment in the five following cognitive domains: attention, language and related functions, visuospatial functions, memory, and frontal/executive functions (Jahng et al., 2015). The SNSB-C is composed of 14 sub-tests, including the Digit Span Test, a shortened version of the Korean-Boston Naming Test, Rey Complex Figure Test, Seoul Verbal Learning Test-Elderly’s version, a shortened version of the Korean-Color Word Stroop Test, Controlled Oral Word Association Test, Korean-Trail Making Test-Elderly’s version, and Digit Symbol Coding (Jahng et al., 2015).

The composite scores of the SNSB-C were expressed as z-scores standardized for age, sex, and education. The score provides an index of overall cognitive functioning, and is an alternative to the Korean Mini-Mental State Examination (which is a brief global instrument used to assess cognitive abilities) for screening patients with cognitive impairment (Jahng et al., 2015). Participants were divided into normal cognition and MCI groups (Kang and Na, 2003). Individuals with normal cognition were defined as those who had a percentile >16th in all sub-tests of the SNSB-C, while individuals with MCI were defined as those with a percentile ≤16th in one or more sub-tests of the SNSB-C.

### Demographic and clinical variables

Demographic variables included age, sex (male or female), and years of education. Medical conditions were assessed based on the presence of hypertension and dyslipidemia. To measure functional disability, a Korean version of the Instrumental Activities of Daily Living (K-IADL) was administered to all participants.

### Statistical analysis

Continuous variables (i.e., age, education years, SNSB scores, and EEG and gait parameters) were compared between the normal cognition and MCI groups using *t*-tests. Between-group comparisons in categorical variables (i.e., sex and the presence of hypertension and/or dyslipidemia) were made using the Chi-squared test. To determine the effect of EEG and gait parameters on cognitive status, we applied logistic regression analysis to estimate the odds ratio (OR) and its 95% confidence interval (CI) for MCI. Linear regression analysis was conducted to estimate beta coefficients and standard error (SE) of the percentile of total SNSB-C scores associated with EEG and gait parameters. These regression models were adjusted for age, sex, education, and medical history of hypertension and dyslipidemia.

To evaluate the diagnostic usefulness of multimodal signals in discriminating between normal cognition and MCI groups, we conducted receiver operating characteristic (ROC) curve analysis. The ROC curve is a plot of sensitivity against 1-specificity for a given diagnostic test. We calculated the area under the ROC curve (AUC), which is a popular indicator of the overall performance of a diagnostic test. The AUC varies from 0.5 (no discrimination, that is, no ability to diagnose individuals with normal and mildly impaired cognition) to 1 (perfect discrimination). We considered three different ROC curves, as follows: (1) including the EEG parameter only, (2) including the gait parameter only, and (3) including both EEG and gait parameters, and provided the AUC value of each.

All analyses were performed using the Statistical Analysis System version 9.2 (SAS Institute, Cary, NC, United States), and statistical significance was set at *p* ≤ 0.05.

## Results

Of the 220 study population, 151 (68.7%) had MCI and 69 had normal cognition at the time of enrollment (Table 1). The mean age of the study participants was 74.2 years, and 54.5% were male. The proportion of individuals with dyslipidemia was the highest at 57.3%, followed by those with hypertension (54.1%) and diabetes mellitus (36.4%). We compared the characteristics between individuals without MCI and those with MCI. There were no significant differences in all characteristics: age (73.1 vs. 74.7 years); period of education (10.3 vs. 10.3 years); proportion of men (52.2 vs. 55.6%); proportion with a history of hypertension (47.8 vs. 56.9%), dyslipidemia (63.8 vs. 54.3%), and diabetes mellitus (44.9 vs. 32.5%); and the K-IADL score (0.1 vs. 0.1). To evaluate the association of EEG and gait parameters with cognitive status, logistic regression (in which MCI was an event) and linear regression (in which the z-score percentile of the total SNSB-C score was a continuous variable) analyses were conducted. The regression models were adjusted for age, sex, years of education, and history of hypertension and/or dyslipidemia. The results are presented in Tables 2–4.

**TABLE 1:** Characteristics of study population according to cognitive impairment status.

Variables	All population	Individuals with normal cognition (*N* = 69)	Individuals with MCI (*N* = 151)	*P*-value
Age (year), mean ± SD	74.2 ± 5.8	73.1 ± 5.1	74.7 ± 6.1	0.0527
Education (year), mean ± SD	10.3 ± 4.5	10.3 ± 4.4	10.3 ± 4.6	0.9172
Male sex, no. (%)	120 (54.5)	36 (52.2)	84 (55.6)	0.6330
Hypertension, no. (%)	119 (54.1)	33 (47.8)	86 (56.9)	0.2075
Dyslipidemia, no. (%)	126 (57.3)	44 (63.8)	82 (54.3)	0.1880
Diabetes mellitus (%)	80 (36.4)	31 (38.8)	49 (61.3)	0.0743
K-IADL, mean ± SD	0.1 ± 0.1	0.1 ± 0.1	0.1 ± 0.1	0.4011

MCI, mild cognitive impairment; K-IADL, Korean version of the Instrumental Activities of Daily Living.

**TABLE 2:** Results from the logistic and linear regression models: OR (95% CI) for MCI and beta coefficients (SE) for SNSB-C score percentile, associated with EEG parameters in relative power.

EEG parameters	Logistic regression model			Linear regression model		
	OR*	(95% CI)	*P*-value	Beta coefficient*	(SE)	*P*-value
**Relative power band**						
Alpha (8–12 Hz)	0.68	(0.24–1.97)	0.4823	11.46	(6.96)	0.1012
Beta (12–25 Hz)	0.27	(0.07–1.00)	0.0507	9.16	(8.97)	0.3082
High beta (25–30 Hz)	0.10	(0.03–0.35)	0.0003	21.97	(7.45)	0.0036
Delta (1–4 Hz)	2.56	(0.39–16.62)	0.3240	−4.80	(12.73)	0.7068
Gamma (30–40 Hz)	0.15	(0.05–0.48)	0.0012	18.29	(6.84)	0.0081
Theta (4–8 Hz)	2.53	(0.74–8.71)	0.1399	−10.78	(7.52)	0.1531

MCI, mild cognitive impairment; SNSB-C, Seoul Neuropsychological Screening Battery-core. *Adjusted for age, sex, education year, and a history of hypertension, dyslipidemia, and diabetes mellitus.

**TABLE 3:** Results from the logistic regression models: OR (95% CI)* for MCI, associated with gait parameters.

Gait parameters	6 m-walking test			10 m-walking test		
	OR*	(95% CI)	*P*-value	OR*	(95% CI)	*P*-value
**Stance phase**						
Hip abduction (degree)	0.93	(0.84–1.03)	0.1545	0.94	(0.83–1.06)	0.2893
Hip flexion (degree)	0.99	(0.94–1.04)	0.6914	0.98	(0.92–1.03)	0.4104
Hip rotation (degree)	0.95	(0.88–1.03)	0.2336	0.90	(0.81–1.00)	0.0475
Knee flexion (degree)	1.00	(0.96–1.05)	0.9536	1.00	(0.95–1.04)	0.8896
Ankle abduction (degree)	0.96	(0.89–1.03)	0.2200	0.92	(0.84–1.00)	0.0534
Ankle dorsiflexion (degree)	0.99	(0.90–1.09)	0.8386	0.98	(0.89–1.08)	0.6688
Ankle inversion (degree)	0.95	(0.88–1.01)	0.0936	0.95	(0.87–1.03)	0.2154
Pelvic tilt (degree)	0.95	(0.75–1.21)	0.6697	0.99	(0.78–1.24)	0.8965
Pelvic obliquity (degree)	0.72	(0.57–0.90)	0.0049	0.72	(0.57–0.91)	0.0055
Pelvic rotation (degree)	0.92	(0.83–1.02)	0.1070	0.96	(0.85–1.07)	0.4411
**Swing phase**						
Hip abduction (degree)	0.92	(0.83–1.03)	0.1652	1.03	(0.88–1.21)	0.7205
Hip flexion (degree)	0.99	(0.93–1.04)	0.6174	0.96	(0.91–1.02)	0.1966
Hip rotation (degree)	0.97	(0.89–1.06)	0.4804	0.94	(0.85–1.04)	0.2352
Knee flexion (degree)	0.99	(0.95–1.03)	0.5107	0.99	(0.95–1.03)	0.6327
Ankle abduction (degree)	0.94	(0.86–1.02)	0.1429	0.93	(0.84–1.02)	0.1366
Ankle dorsiflexion (degree)	0.87	(0.78–0.97)	0.0119	0.88	(0.80–0.98)	0.0145
Ankle inversion (degree)	0.95	(0.89–1.01)	0.1038	0.92	(0.84–1.01)	0.0709
Pelvic tilt (degree)	0.86	(0.69–1.08)	0.2044	0.94	(0.70–1.25)	0.6654
Pelvic obliquity (degree)	0.51	(0.35–0.76)	0.0008	0.51	(0.34–0.77)	0.0016
Pelvic rotation (degree)	0.90	(0.82–1.00)	0.0503	0.95	(0.84–1.07)	0.3943

MCI, mild cognitive impairment; SNSB-C, Seoul Neuropsychological Screening Battery-core. *Adjusted for age, sex, education year, and a history of hypertension, dyslipidemia, and diabetes mellitus.

**TABLE 4:** Results from the linear regression models: beta coefficients (SE)* for SNSB-C score percentile, associated with gait parameters.

Gait parameters	6 m-walking test			10 m-walking test		
	Beta coefficient*	(SE)	*P*-value	Beta coefficient*	(SE)	*P*-value
**Stance phase**						
Hip abduction (degree)	1.19	(0.67)	0.0772	0.84	(0.81)	0.3029
Hip flexion (degree)	0.04	(0.32)	0.8919	0.19	(0.36)	0.6012
Hip rotation (degree)	0.73	(0.54)	0.1795	0.42	(0.65)	0.517
Knee flexion (degree)	0.29	(0.29)	0.3232	0.11	(0.30)	0.7134
Ankle abduction (degree)	1.34	(0.49)	0.0066	1.41	(0.57)	0.0136
Ankle dorsiflexion (degree)	−0.45	(0.65)	0.4829	−0.41	(0.61)	0.5054
Ankle inversion (degree)	0.96	(0.43)	0.0278	0.47	(0.55)	0.3984
Pelvic tilt (degree)	1.71	(1.58)	0.2817	2.13	(1.49)	0.1565
Pelvic obliquity (degree)	4.59	(1.41)	0.0013	4.68	(1.41)	0.0010
Pelvic rotation (degree)	1.34	(0.66)	0.0446	0.79	(0.74)	0.2887
**Swing phase**						
Hip abduction (degree)	1.46	(0.74)	0.0482	0.59	(1.06)	0.5751
Hip flexion (degree)	0.21	(0.37)	0.5737	0.55	(0.38)	0.1495
Hip rotation (degree)	0.71	(0.55)	0.1980	0.08	(0.65)	0.8976
Knee flexion (degree)	0.53	(0.27)	0.0548	0.56	(0.28)	0.0464
Ankle abduction (degree)	1.61	(0.54)	0.0033	1.52	(0.64)	0.0185
Ankle dorsiflexion (degree)	1.10	(0.69)	0.1123	0.78	(0.65)	0.2325
Ankle inversion (degree)	1.00	(0.44)	0.0249	0.61	(0.59)	0.3012
Pelvic tilt (degree)	0.77	(1.50)	0.6097	2.88	(1.91)	0.1329
Pelvic obliquity (degree)	9.43	(2.29)	<0.0001	9.97	(2.53)	0.0001
Pelvic rotation (degree)	1.54	(0.68)	0.0239	1.09	(0.80)	0.1727

*Adjusted for age, sex, education year, and a history of hypertension, dyslipidemia, and diabetes mellitus.

For the relative power of EEG parameters (Table 2), ORs (95% CI) for MCI were significantly associated with decreases in high beta (adjusted OR = 0.10; 95% CI = 0.03–0.35, *p* = 0.0003) and gamma (adjusted OR = 0.15; 95% CI = 0.05–0.48, *p* = 0.0012). In the linear regression, beta coefficients (SE) for the SNSB-C score were significantly associated with increases in high beta (adjusted beta = 21.97; SE = 7.45, *p* = 0.0036) and gamma bands (adjusted beta = 18.29; SE = 6.84, *p* = 0.0081) in relative power.

For gait parameters (Table 3), a decrease in pelvic obliquity angle (6-m walking: adjusted OR = 0.72; 95% CI = 0.57–0.90, *p* = 0.0049; 10-m walking: adjusted OR = 0.72; 95% CI = 0.57–0.91, *p* = 0.0055) during the stance phase and decreases in ankle dorsiflexion angle (6-m walking: adjusted OR = 0.87; 95% CI = 0.78–0.97, *p* = 0.0119; 10-m walking: adjusted OR = 0.88; 95% CI = 0.80–0.98, *p* = 0.0145) and pelvic obliquity angle (6-m walking: adjusted OR = 0.51; 95% CI = 0.35–0.76, *p* = 0.0008; 10-m walking: adjusted OR = 0.51; 95% CI = 0.34–0.77, *p* = 0.0016) during the swing phase consistently and significantly increased the likelihood for developing MCI in both the 6- and 10-m walking tests.

The linear regression (Table 4) of kinematic gait parameters revealed that beta coefficients (SE) for the SNSB-C score consistently increased in terms of increases in ankle abduction angle and pelvic obliquity angle during both the 6- and 10-m walking tests. In the stance phase, adjusted beta coefficients (SE) during the 6- and 10-m walking tests were 1.34 (0.49; *p* = 0.0066) and 1.41 (0.57; *p* = 0.0136) for ankle abduction angle, and 4.59 (1.41; *p* = 0.0013) and 4.68 (1.41; *p* = 0.0010) for pelvic obliquity angle. In the swing phase, adjusted beta coefficients (SE) during the 6- and 10-m walking tests were 1.61 (0.54; *p* = 0.0033) and 1.52 (0.64; *p* = 0.0185) for ankle abduction angle, and 9.43 (2.29; *p* < 0.0001) and 9.97 (2.53; *p* = 0.0001) for pelvic obliquity angle.

Based on the chosen significance level of α, high-frequency EEG activity in high beta (25–30 Hz) and gamma (30–40 Hz) bands was significantly associated with MCI and cognitive performance. Among the gait parameters, the pelvic obliquity angle in kinematics was significantly associated with MCI and cognitive performance, and this association was consistent in both the 6- and 10-m walking tests.

**FIGURE 1:**
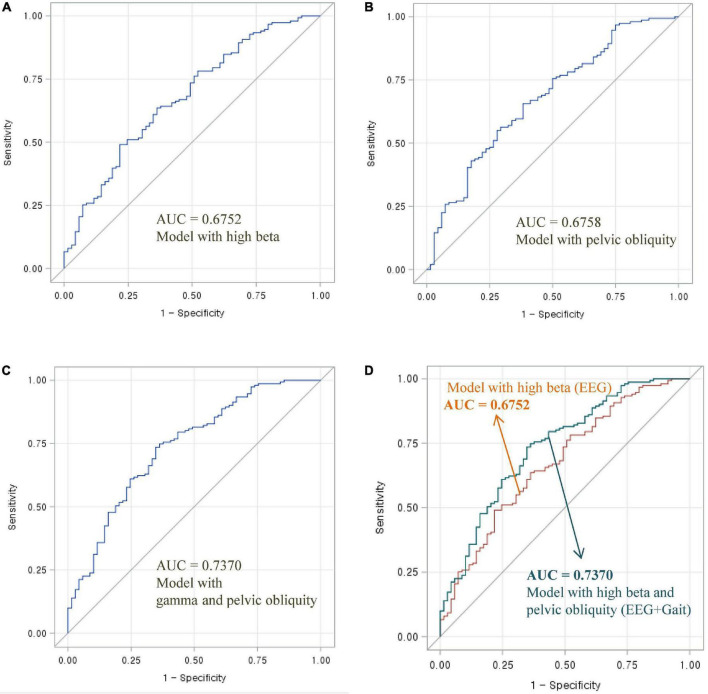
Area under the ROC curve of EEG and gait parameters for classifying MCI and normal cognition: **(A)** Model with high beta power, **(B)** model with pelvic oblique, **(C)** combined model with high beta power and pelvic oblique, and **(D)** comparison of AUC curves.

**FIGURE 2:**
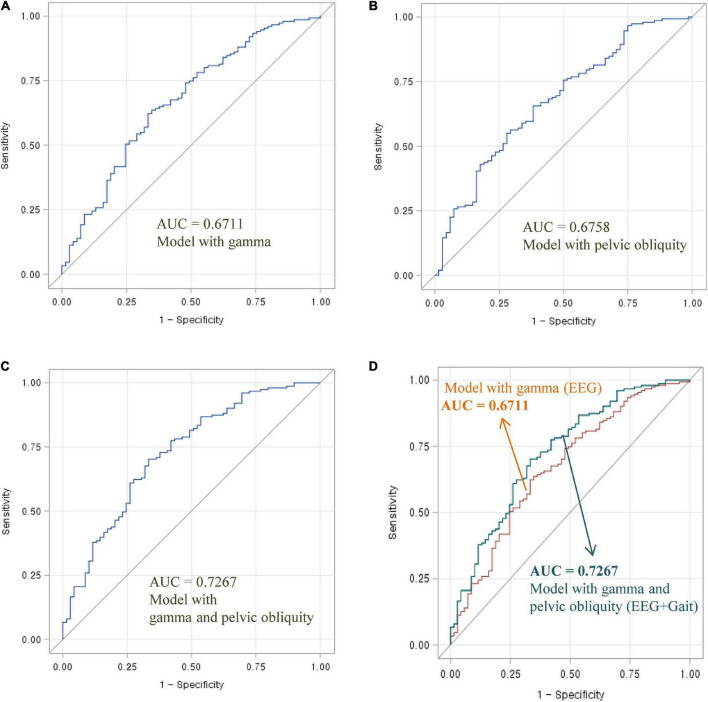
Area under the ROC curve of EEG and gait parameters for classifying MCI and normal cognition: **(A)** Model with gamma power, **(B)** model with pelvic oblique, **(C)** combined model with gamma power and pelvic oblique, and **(D)** comparison of AUC curves.

To assess the diagnostic utility of multimodal signals (i.e., the combination of EEG and gait parameters) for distinguishing normal and mildly impaired cognition, ROC curves and AUC values were used (Figures 1, 2). The three following ROC curves were created and calculated: (A) model with EEG parameters (high beta or gamma), (B) model with gait parameters (pelvic obliquity angle), and (C) model with EEG and gait parameters (high beta with pelvic obliquity or gamma with pelvic obliquity).

As shown in Figure 1, the AUC value was 0.6752 for high beta activity (A) and 0.6758 for pelvic obliquity angle (B). Calculation of the AUC demonstrated better performance (AUC = 0.7370) by the combination of the two parameters (D) than by a single parameter (either high beta or pelvic obliquity). As shown in Figure 2, the AUC value was 0.6711 for gamma activity (A) and 0.6758 for pelvic obliquity angle (B). Calculation of the AUC demonstrated better performance (AUC = 0.7267) by the combination of the two parameters (D) than by a single parameter (either gamma or pelvic obliquity).

## Discussion

Our results confirmed that specific EEG and gait parameters were significantly associated with cognitive status. Individuals with decreases in high-frequency EEG activity in high beta (25–30 Hz) and gamma (30–40 Hz) bands increased the probability of MCI. We also found an association between the pelvic obliquity angle and cognitive status, assessed by MCI or SNSB-C scores. The main aim of this study was to determine whether multimodal signals with EEG and gait parameters are meaningful in distinguishing cognitive impairment. Results from the ROC analysis revealed that multimodal signals combining high beta or gamma and pelvic obliquity improved the ability to discriminate MCI individuals from normal controls. These findings support prior work on the association between cognitive status and EEG or gait, and offer new insights into the applicability of multimodal signals to distinguish cognitive impairment.

Neural oscillations are rhythmic or repetitive electrical discharges in the brain (Başar, 2013). EEG generates time-series data on neural oscillations in the brain, even though voltage oscillations detected on the scalp are small (Jeong, 2004). We found significant decreases in high beta (25–30 Hz) and gamma (30–40 Hz) band oscillations in the MCI group, as assessed by the SNSB-C. Conversely, increased high beta and gamma activities were significantly associated with better cognitive performance.

Beta activity is typically observed in motor activity (Honaga et al., 2010). Although voltage oscillations detected on the scalp are small, EEG generates time-series data on neural oscillations at different frequency bands (i.e., delta, theta, alpha, beta, and gamma) in the brain (Jeong, 2004). Delta oscillations are prominent in the early developmental stages and during slow-wave sleep (Knyazev, 2012). Functionally, delta oscillations are implicated in the synchronization of brain activity with autonomic functions, motivational processes associated with both reward and atavistic defensive mechanisms, and in cognitive processes mostly related to attention and the detection of motivationally salient stimuli in the environment (Knyazev, 2012). Theta oscillations are low-frequency oscillations in the local field potential within the hippocampus, amygdala, and neocortex (Hutchison and Rathore, 2015). Theta activity is dominant feature synchronized across brain regions during both wake and rapid-eye movement sleep (Hutchison and Rathore, 2015). Alpha oscillations are the most prominent EEG features during wakefulness; the more an individual is stable and relaxed, the greater the amplitude (Halgren et al., 2019). Beta oscillations are typically observed during motor activity (Honaga et al., 2010). The modulation of beta band oscillations plays a key role in action planning and execution, wherein the beta power decreases during action planning and decreases further during action execution. Gamma oscillations are the fastest and subtlest brain waves. Gamma power modulates perception and consciousness (Missonnier et al., 2010). Delta, theta, and alpha oscillations are global processing modes that cover relatively large cortical regions and serve integration across diverse cortical sites by synchronizing coherent activity and phase coupling across widely spatially distributed neural assemblies. Oscillations of beta and gamma ranges or local EEG modes are distributed in higher frequencies, lower amplitudes, and a more limited topographic area (Knyazev, 2012). Although the direction of increase or decrease in each oscillation is not consistent owing to different experimental protocols and participant’ characteristics, a substantial evidence suggests that oscillations in all frequency bands are linked to cognitive processes such as attention, perception, and memory (Missonnier et al., 2007, 2010; Ray and Maunsell, 2015; Spitzer and Haegens, 2017). Our data displayed the association of high beta/gamma oscillations with cognitive performance. Using the SNSB-C, we found significant decreases in high beta (25–30 Hz) and gamma (30–40 Hz) band oscillations in the MCI group. Many studies have shown that a decrease in beta or gamma power is a characteristic of AD progression (Başar and Düzgün, 2016; Wang et al., 2017; Smailovic and Jelic, 2019). There is scarce evidence of any such spectral changes in patients with MCI. Güntekin et al. (2013) found that patients with MCI tended to have a lower beta response than healthy controls during the visual oddball paradigm. Michels et al. (2017) investigated the association between amyloid deposition and ApoE and EEG measurements in 17 healthy controls and 17 patients with MCI. The authors revealed that beta power was lower in patients with MCI relative to healthy controls, and was negatively associated with ApoE4-status and global amyloid deposition (*r* = 0.51, *p* = 0.02) (Michels et al., 2017). In a recent case-control study, Murty et al. (2021) measured patterns of brain activity in elderly people classified as healthy, with MCI, and with AD. They found significantly reduced stimulus-induced gamma rhythms in the patients with MCI and AD compared to age- and sex-matched controls (Murty et al., 2021). Taken together, these studies indicate that high beta and gamma levels are associated with cognitive impairment. While our results can be interpreted in a similar way, the current data does not provide information about the mechanism underlying the association of higher frequency bands, especially high beta and gamma bands, with cognitive performance.

Gait and cognition share anatomical structures and brain control processes (Cohen et al., 2016). Close associations between gait abnormalities and cognitive deficits have been reported in the elderly (Pieruccini-Faria et al., 2021). Consistent with prior work, we also observed a significant difference in certain kinematic gait variables between individuals with normal cognition and those with MCI. There was a strong and consistent association between lower pelvic obliquity angle and cognitive status (assessed by MCI or SNSB-C scores) in both 6- and 10-m walking tests. This indicates that kinematic gait disturbance and cognitive decline may be correlated. Most studies have stressed the importance of spatio-temporal gait features (i.e., slow gait velocity and shorter stride length and time) that can distinguish cognitive impairment (Toots et al., 2019; Xie et al., 2019; Sebastiani et al., 2020). There has been little interest in gait kinematics according to cognitive status; two recent studies have provided evidence of an association between gait kinematics and cognitive status (Rucco et al., 2017; Zhong et al., 2021). Rucco et al. (2017) examined gait patterns in patients with AD and those with behavioral variants of frontotemporal dementia. A significant increase in dorsiflexion and a decrease in plantar flexion under cognitive dual-task conditions were observed in patients with AD relative to the normal controls (Rucco et al., 2017). More recently, Zhong et al. (2021) found that older adults with MCI had larger knee peak extension angles and smaller knee heel strike angles than those with normal cognition. These previous studies suggest that cognitive impairment may affect gait kinematic parameters, along with spatio-temporal parameters. Regarding the relationship between pelvic obliquity and cognitive impairment, the results of Martín-Gonzalo et al. (2019) seem to support our own, showing the discrimination of pelvic movement according to the status of cognitive impairment. The authors compared gait kinematic metrics (complexity and irreversibility) in patients with MCI and mild AD relative to healthy controls. The amount of irreversibility of the pelvic tilt kinematic signal was decreased in patients with MCI and mild AD, whereas complexity in the hip flexion and pelvic obliquity kinematic signals was decreased in patients with mild AD. These results suggest that kinematic disorganization is present at an early stage of cognitive impairment. Further studies are needed to understand how individuals with cognitive decline adapt to the progressive deterioration of motor functions, specifically gait kinematics.

The primary aim of this study was to examine whether combined EEG and gait parameters are useful in distinguishing between cognitive impairment and normal cognition. Previous studies have analyzed the usefulness of multimodal techniques, such as using machine learning methods and combining neuroimaging data, biological tests, or clinical records, in the early diagnosis of cognitive decline and AD. To the best of our knowledge, the present study is the first to use multimodal signals that incorporate EEG and gait to classify MCI risk. As shown in Figures 1, 2, the combination of EEG and gait kinematic parameters (high beta power with pelvic obliquity AUC = 0.7370 and gamma power with pelvic obliquity AUC = 0.7267) had additional value compared to high beta power (AUC = 0.6752), gamma power (AUC = 0.6300), and pelvic obliquity (AUC = 0.6758) alone. This is encouraging, as it indicates that multimodal signals significantly improved MCI prediction compared with predictions made by the models using EEG or gait data only. Given the benefits of EEG and gait as being non-invasive, relatively inexpensive, and easily available measures, our multimodal approach may be effective for screening of cognitive decline and monitoring AD risk. However, this study has several limitations, such as its cross-sectional design, unmeasured confounders, and the possibility of misclassified MCI. Thus, as with any diagnostic test, its clinical utility should be confirmed in follow-up studies.

In conclusion, we found that high-frequency EEG activity and pelvic obliquity angle were significantly associated with cognitive status. This finding supports the proposal that multimodal signals could be used to identify individuals at risk of cognitive impairment. However, further studies to verify the reliability of the combined use of EEG and kinetic gait measures for identifying MCI individuals are needed because these measures need to be contrasted with other, more easily obtained, and reliable predictors of MCI (e.g., gait speed). Additional studies are also required to develop guidelines for using EEG and kinetic gait measures for MCI prediction that can be applied in clinical settings. If our follow-up study reveals that multimodal signals combining EEG and gait are able to reasonably predict MCI risk, this might be a good strategy to improve the performance of MCI diagnostic tests.

## Data availability statement

The original contributions presented in this study are included in the article/supplementary material, further inquiries can be directed to the corresponding author.

## Ethics statement

The studies involving human participants were reviewed and approved by the Institutional Ethical Review Board of the Veterans Health Service Medical Center (IRB no. BOHUN 2021-02-024-002). The patients/participants provided their written informed consent to participate in this study.

## Author contributions

J-YM planned the study, performed the statistical analyses, and wrote the manuscript. S-WH supervised the data collection and analysis and revised the manuscript. KL reviewed the collected data and revised the manuscript. K-BM received funding, planned the study, supervised the data collection and analysis, and contributed to manuscript. All authors contributed to the article and approved the submitted version.
