# Corrigendum: Use of electroencephalogram, gait, and their combined signals for classifying cognitive impairment and normal cognition

**DOI:** 10.3389/fnagi.2023.1349594

**Published:** 2023-12-20

**Authors:** Jin-Young Min, Sang-Won Ha, Kiwon Lee, Kyoung-Bok Min

**Affiliations:** ^1^Veterans Medical Research Institute, Veterans Health Service Medical Center, Seoul, South Korea; ^2^Department of Neurology, Veterans Health Service Medical Center, Seoul, South Korea; ^3^Ybrain Research Institute, Seongnam-si, South Korea; ^4^Department of Preventive Medicine, College of Medicine, Seoul National University, Seoul, South Korea; ^5^Medical Research Center, Institute of Health Policy and Management, Seoul National University, Seoul, South Korea

**Keywords:** diagnosis, cognitive impairment, multimodal signals, EEG, kinematic gait analysis

In the published article, there was an error.

A correction has been made to **Methods**, *study population*.

This sentence previously stated:

“Individuals aged ≥60 years who visited the Department of Neurology at Veterans Health Service Medical Center (Seoul, South Korea) between January and September 2021 were the target population for the current study.”

The corrected sentence appears below:

“Individuals aged ≥60 years who visited the Department of Neurology at Veterans Health Service Medical Center (Seoul, South Korea) between March and December 2021 were the target population for the current study.”

A correction has been made to **Methods**, *study population*.

This sentence previously stated:

“The study protocols were approved by the Institutional Ethical Review Board of the Veterans Health Service Medical Center (IRB no. BOHUN 2021-02-024-001, BOHUN 2021-01-066-006).”

The corrected sentence appears below:

“The study protocols were approved by the Institutional Ethical Review Board of the Veterans Health Service Medical Center (IRB No. BOHUN 2021-02-024).”

The authors apologize for this error and state that this does not change the scientific conclusions of the article in any way. The original article has been updated.

